# Supplementary material for: FERN – a Java framework for stochastic simulation and evaluation of reaction networks
Source: BMC Bioinformatics. 2008 Aug 29;9:356. doi: 10.1186/1471-2105-9-356 (PMC2553347; doi:10.1186/1471-2105-9-356)
Supplement: Additional file 1 — FERN distribution, Version 1.3. This archive contains the FERN source code and binaries as well as documentation and example models in FernML and SBML. [file 1471-2105-9-356-S1.zip › fern/doc/javadoc/fern/network/sbml/MathTree.ConstLeaf.html]

MathTree.ConstLeaf


---


|  |  |  |
| --- | --- | --- |
| **Overview** \| **Package** \| **Class** \| **Use** \| **Tree** \| **Deprecated** \| **Index** \| **Help** | |  |
| **PREV CLASS**   **NEXT CLASS** | **FRAMES**    **NO FRAMES**     **All Classes** |  |
| SUMMARY: NESTED \| FIELD \| CONSTR \| METHOD | DETAIL: FIELD \| CONSTR \| METHOD |  |


---


## fern.network.sbml Class MathTree.ConstLeaf

```
java.lang.Object
  fern.network.sbml.MathTree.Node
      fern.network.sbml.MathTree.ConstLeaf
```

**Enclosing class:**: MathTree

---

``` public static class MathTree.ConstLeaf extends MathTree.Node ```

---

| **Field Summary** | |
| --- | --- |
| `double` | `Value` |


**Constructor Summary**

`MathTree.ConstLeaf(double Value)`


| **Method Summary** | |
| --- | --- |
| `String` | `toString()` |

**Methods inherited from class java.lang.Object**

`clone, equals, finalize, getClass, hashCode, notify, notifyAll, wait, wait, wait`

**Field Detail**

### Value

```
public double Value
```


**Constructor Detail**

### MathTree.ConstLeaf

```
public MathTree.ConstLeaf(double Value)
```


**Method Detail**

### toString

```
public String toString()
```

:   **Overrides:**: `toString` in class `Object`


---


|  |  |  |
| --- | --- | --- |
| **Overview** \| **Package** \| **Class** \| **Use** \| **Tree** \| **Deprecated** \| **Index** \| **Help** | |  |
| **PREV CLASS**   **NEXT CLASS** | **FRAMES**    **NO FRAMES**     **All Classes** |  |
| SUMMARY: NESTED \| FIELD \| CONSTR \| METHOD | DETAIL: FIELD \| CONSTR \| METHOD |  |


---
